# Evaluation of prediction effect of perfusion index for supraclavicular brachial plexus block in children: protocol for a randomized trial

**DOI:** 10.1186/s13063-022-06597-y

**Published:** 2022-08-04

**Authors:** Jinxu Wang, Lingli Deng, Aijun Xu

**Affiliations:** Department of Anesthesiology and Pain Medicine, Tongji Hospital, Huazhong University of Science and Technology, 1095 Jiefang Avenue, Wuhan, 430030 China

**Keywords:** Perfusion Index, Supraclavicular brachial plexus block, Anesthesia, Pediatric

## Abstract

**Background:**

Pulse perfusion index (PI) reflects blood perfusion. It has been reported that PI can be used to evaluate the effect of nerve block, but currently, it is mainly focused on awake adults. In pediatric general anesthesia, it has been reported that PI can evaluate the effect of the sacral block. Still, there is a lack of relevant research on the impact of brachial plexus blocks. Our objective is to assess the prediction effects of PI on the success of supraclavicular brachial plexus block in pediatric patients under sevoflurane or propofol general anesthesia.

**Methods/design:**

This is a mono-center, parallel, 2-arm randomized superiority trial. One hundred four children aged 1 month to 12 years who undergo upper limb surgery will be enrolled in this study. According to anesthesia induction and maintenance medication, they will be divided into sevoflurane and propofol groups. The PI values of the index and little finger will be recorded on the blocked and non-blocked sides of supraclavicular brachial plexus block (SCB) in all children. The primary outcome is to assess the effects of PI on the success of supraclavicular brachial plexus block in pediatric patients under sevoflurane or propofol general anesthesia. The secondary outcome includes mean arterial blood pressure (MAP), heart rate (HR), and correlation between baseline PI and 10 min after SCB (PI ratio).

**Discussion:**

This trial will provide evidence on the changes in PI after SCB in sevoflurane or propofol anesthesia in children. SCB may lead to changes in PI values under sevoflurane or propofol anesthesia. After the children wake up at the end of the surgery, the changes in PI values on the block side and non-block side may be helpful to judge the effect of nerve block when excluding the influence of anesthetics.

**Trial registration:**

ClinicalTrials.gov NCT04216823. Registered on 15 July 2020.

## Introduction

Fractures are common diseases in the growth and development of children [1]. Naranje SM et al. reported that the incidence of fractures is 180/1000 throughout childhood and adolescence, and 17.8% of all fractures is forearm fracture. Finger and wrist fractures are the second and third most common fractures [2]. An epidemiological analysis of 1067 child fractures in Switzerland indicated that 76% of the fractures were upper limb fractures. Among them, 86% (694) were treated with plaster fixation or closed reduction, 11% (92) were treated with fast reduction nails or elastic stable intramedullary nails, and 3% required open reduction and internal fixation [3]. In addition, fractures cause movement limitation and obvious pain. Fully adequate anesthesia and analgesia are essential parts of fracture treatment.

Brachial plexus block (BCB) has a long history of being used for anesthesia and postoperative analgesia in upper limb surgery [4, 5]. There are four frequently used approaches for brachial plexus block: interscalene block, supraclavicular block, subclavian block, and axillary block [6, 7]. Traditionally, the supraclavicular block is seldom used because it is prone to pneumothorax, phrenic nerve block, intravascular injection, and Horner syndrome [8]. In recent years, with the development of real-time ultrasound-guided nerve blocks, anesthesiologists with extensive experience in ultrasound-guided nerve blocks can safely perform supraclavicular brachial plexus block in children under general anesthesia [9, 10]. Most regional anesthesia is performed under general anesthesia because uncooperative children may increase the risk of additional injury [11]. Walker BJ et al. analyzed the data of 100,000 children with regional block from more than 20 hospitals. They believed that regional anesthesia under general anesthesia was safe, and none of the children had permanent nerve damage, which was consistent with the risk of nerve block in awake adult patients [12]. However, evaluating the effect of nerve block usually requires the patient's cooperation, and it is unsuitable for patients under sedation and general anesthesia. In recent years, some scholars have discovered new objective indicators for estimating the effect of nerve block [13]. The pulse perfusion index (PI) is an indicator that reflects blood perfusion [14]. It is the pulsating blood flow to non-pulsating blood flow in peripheral tissues measured by a special pulse oximeter and is regulated by autonomic nerves. After a successful nerve block, the blocked autonomic nerve caused local vascular dilatation. It increases skin temperature, as well as increases regional perfusion, which will lead to changes in PI value [15].

Currently, studies found that PI can be used to evaluate the effect of nerve block primarily focused on awake adults [16, 17]. Xu Z reported that PI provides an earlier and more sensitive indicator to assess the onset of caudal block under ketamine anesthesia [18]. Still, there is a lack of relevant research on the effect of peripheral nerve block in children. The supraclavicular brachial plexus block (SCB) can be used for surgical anesthesia and analgesia of the upper arm, elbow, forearm, wrist, and hand below the shoulder joint. Therefore, the aim of this study is to evaluate the effect of PI on predicting the success of SCB in children under general anesthesia with sevoflurane and propofol.

## Methods and design

### Aim of the study

This study aims to describe the role of PI in evaluating the predictive effect of SCB in children under general anesthesia with sevoflurane and propofol. Meanwhile, whether PI could predict the success of SCB by reflecting the impact of ulnar nerve block.

### Design of the study

**Fig. 1:**
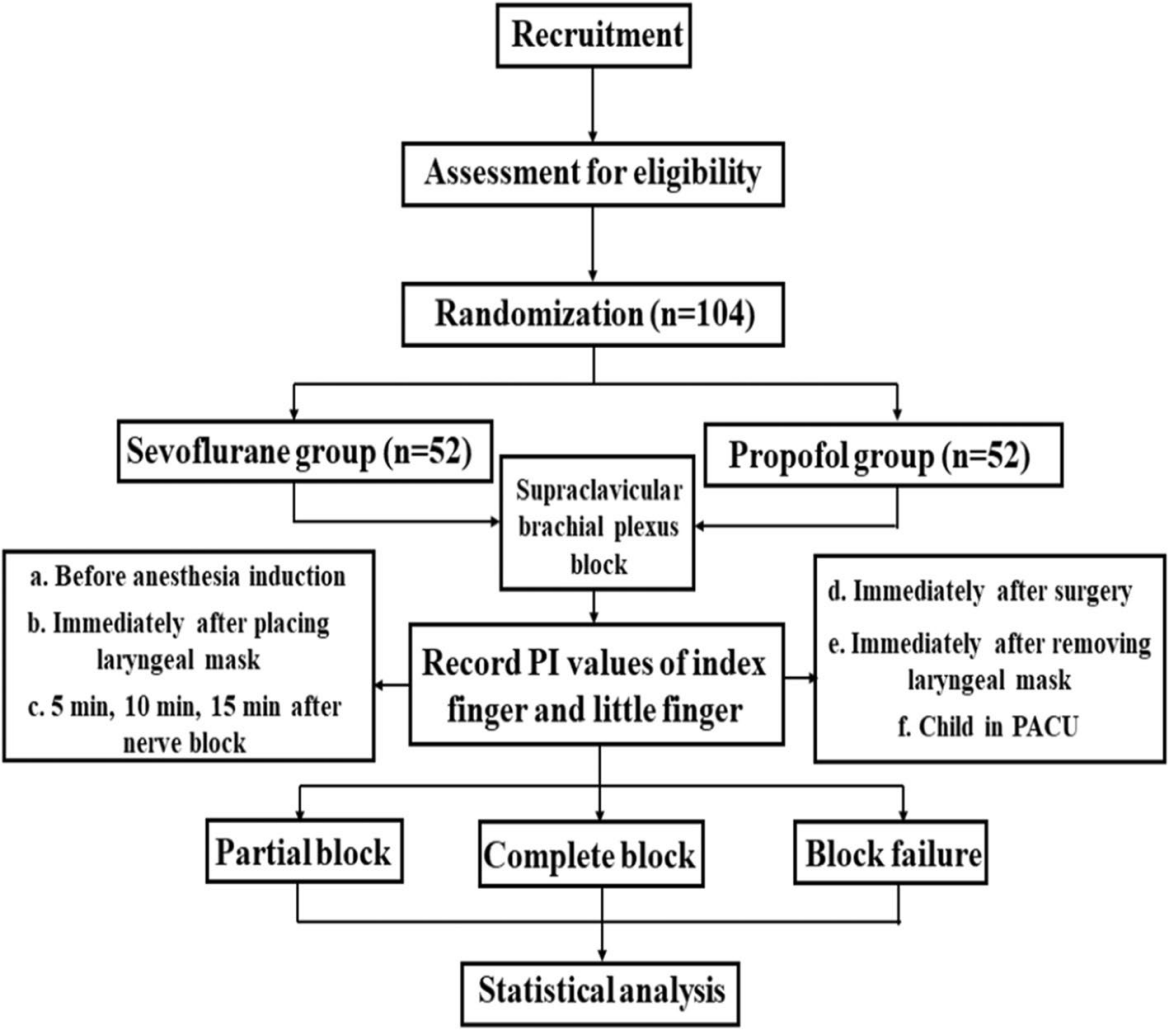
Flowchart of this study

**Fig. 2:**
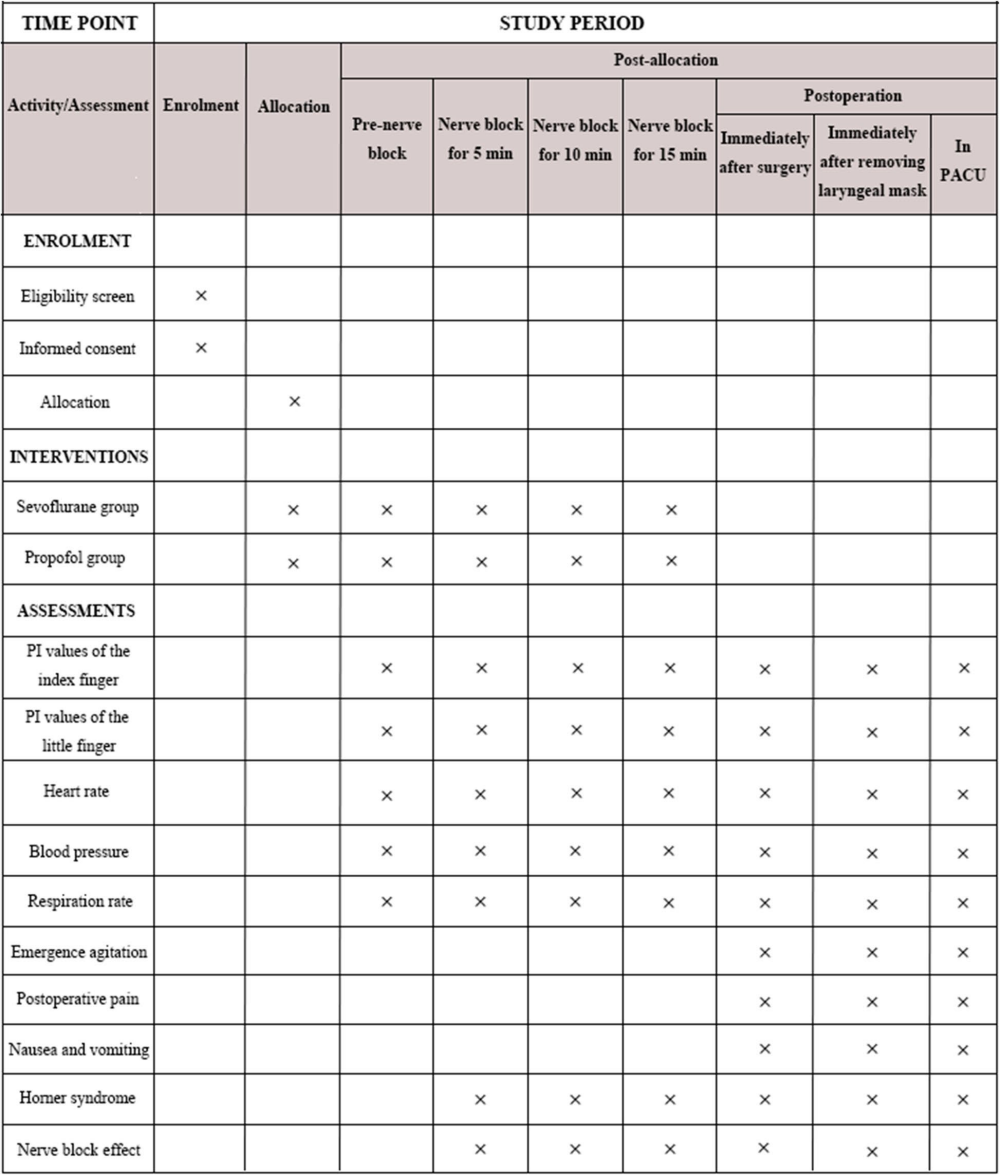
Participant timeline

This is a parallel, 2-arm randomized superiority trial (protocol version 1.0, 05.22.2020). Tongji Hospital affiliated to Tongji Medical College of Huazhong University of Science & Technology acting as the sponsor. Pediatric upper limb surgery cases will be collected in Tongji Hospital from May 2020 to December 2022. Children were divided into propofol and sevoflurane groups by the random number table method. PI values of the index finger and little finger in the blocked and non-blocked sides will be recorded in all children. Analyzing the possible relationship of the changes in PI values with complete block, partial block, and block failure in each group. A comparative analysis of sevoflurane and propofol block cases will determine whether PI can better judge the effect of supraclavicular brachial plexus block in which general anesthesia in children. The flowchart of this study is presented in Fig. 1. The schedule of enrollment, interventions, and assessments is shown in Fig. 2.

### Sample size

According to literature reports [15], the probability of ulnar nerve insufficiency is less than 20%; the sample size is calculated: As shown in Table 1, the preliminary estimated sample size is 47 cases, and the withdrawal rate is assumed to be 10%. The calculation is 52 cases per group, so 104 children will be included in this experiment.

**Table 1:** Sample size estimation results

		Type I Error- Alpha			
		0.20	0.10	0.05	0.01
Type II Error -Beta	0.20	16 + 4	23 + 6	30 + 8	46 + 12
	0.10	21 + 6	29 + 8	**37 + 10**	54 + 14
	0.05	26 + 7	35 + 9	43 + 11	62 + 16
	0.01	37 + 10	47 + 12	57 + 15	78 + 20

### Eligibility and exclusion criteria

Eligibility criteria:

1. Age from 1 month to 12 years
2. ASA class I–II
3. Elective upper extremity surgery in children
4. Sign the informed consent, and agree to participate

Exclusion criteria:

1. Refusal by family members
2. Children with abnormal behavior, including internalizing problems and externalizing problems
3. Inflection at the puncture site
4. Other regional anesthesia contraindications
5. Children are allergic to known ingredients of experimental drugs

Withdrawal criteria:

1. After selection, the patient is found not to meet the selection or exclusion criteria.
2. Children experienced severe adverse reactions or exacerbations during the clinical study.

### Randomization

Prior to the commencement of the clinical study, the investigator will discuss the trial with the children and children’s guardian, including the research nature, the purpose of the study, possible benefits and risks, and the rights and obligations of the subject. The guardian signed the consent form, and the children who meet the eligibility criteria were randomly assigned to the sevoflurane group (SEV group) or propofol group (PRO group) in a 1:1 allocation. A computer-generated randomization schedule will allocate subjects. Allocations were maintained on a secure website accessed just before the procedure begin.

### Implementation

After screening the child as a qualified subject and giving informed consent, a sealed opaque envelope containing a card with the computer-generated assignment number (1 = SEV group, 2 = PRO group) will be opened by an anesthesia assistant who was not involved in the study. Anesthesiologists unblinded to the grouping of subjects are responsible for anesthesia management and nerve block according to the predetermined anesthesia.

### Interventions

The children will be given a slow intravenous bolus of 0.1 mg/kg midazolam for sedation during the waiting period. After entering the operating room, routinely monitor the electrocardiogram (ECG), heart rate (HR), blood pressure (BP), respiration rate (RR), and pulse oxygen saturation (S_P_O_2_). SEV group: Inhalation of sevoflurane (5%-8%) for induction of anesthesia, sufentanil 0.2 μg/kg, penehyclidine hydrochloride 0.01mg/kg, dexamethasone 0.1 mg/kg. Place the laryngeal mask, and maintain anesthesia with 1.0 MAC value of sevoflurane + 50% oxygen. PRO group: propofol 3 mg/kg, sufentanil 0.2 μg/kg, penehyclidine hydrochloride 0.01 mg/kg, dexamethasone 0.1 mg/kg are given for anesthesia induction. Place the laryngeal mask, and propofol 3 mg/kg/h is given for anesthesia maintenance. Then, all children will be placed supine with their heads tilted to the opposite side. Under ultrasound guidance, an experienced anesthesiologist will use an in-plane needle to block the supraclavicular nerve and give 0.25% ropivacaine 0.4 ml/kg.

### Outcomes

#### Primary outcome

The primary outcome is to assess the effects of PI on the success of supraclavicular brachial plexus block in pediatric patients under sevoflurane or propofol general anesthesia. PI values of the index finger and little finger will be recorded before induction of general anesthesia in the operating room, immediately after laryngeal mask placement, 5 min, 10 min, 15 min after SCB, immediately after surgery, after removal of laryngeal mask when the child is awake and in PACU. Record the dosages of opioids and vasoactive drugs during the operation. Evaluate the complications and recovery quality in children. (1) Assessment of restlessness and delirium during the recovery period and 30 min in PACU. (2) Postoperative pain score. Postoperative complications: nausea and vomiting, neurological dysfunction, Horner syndrome, respiratory depression, etc.

#### Secondary outcomes

The secondary outcome includes mean arterial blood pressure (MAP), heart rate (HR), and PI ratio (correlation between baseline PI and 10min after SCB). The age, gender, ASA classification, BMI, anesthesia time, operation time, anesthesia recovery time, duration of stay in PACU, and duration of postoperative hospital stay will be recorded.

### Block effect evaluation

Complete block: There is no significant fluctuation of the HR, BP, and RR during the operation (heart rate and blood pressure increased by less than 10% compared with the baseline value, and respiratory rate increased by less than 20%). Partial block: When a non-single nerve innervates the surgical site, there is a specific innervated area that causes HR, BP, and RR fluctuations (HR, BP increase ≥ 10% from the baseline value, RR increase ≥ 20%), while there is no significant fluctuation in operation in other innervated area. Block failure: Operation in any innervated area caused substantial changes in HR, BP, and RR. For partial block and failure of block, a single dose of sufentanil (0.01 μg/kg) and a pump of 0.1–0.2 μg/kg/min remifentanil for supplementary analgesia.

### Data collection and management

Record the PI values of the index finger and little finger on blocked and non-blocked sides in all children. Data collection and completion will be carried out by two research assistants blinded to the grouping of subjects. The clinical trial supervisors will periodically review the quality of data entry to ensure the authenticity and reliability of the program. This data supervision committee has no conflict of interest in the study. All information related to the study will be stored securely.

### Statistical analyses

Analyses will be performed using SPSS statistical software (version 19.0). Categorical variables are represented by frequency, and continuous variables are represented by mean ± standard deviation or median [interquartile range]. Chi-square test or Fisher test will be used for comparison between groups of classified variables, and *t*-test, analysis of variance, or rank-sum test will be used for comparison between groups of continuous variables. A logistic regression model will be used to analyze the influencing factors of anesthesia effect and complications, and stepwise regression will be used to screen independent variables. If data were missing, patients would be excluded from the specific analysis.

### Auditing

There is no audit in the study.

### Possible benefits of participation

All children undergoing ultrasound-guided supraclavicular brachial plexus block for anesthesia can improve postoperative pain, reduce the use of postoperative analgesics, and promote recovery. In addition, the study will also benefit children undergoing upper limb surgery in the future.

### Ancillary and post-trial care

This study is observational. The possible adverse risks are all inherent complications and risks in surgery and anesthesia, and no additional adverse reactions and risks are added. Once any risk of harm is identified, the principal investigator will intervene to minimize potential harm. If there is any damage, the research team will provide appropriate compensation if necessary.

### Protocol amendments

Any possible protocol modification during the trial will require formal protocol modification. The Ethics Committee will conduct a comprehensive assessment and review of the subjects’ risks and inform all members after the new protocol is approved.

### Dissemination policy

The trial results will be published in the Clinical Trial Registry database or presented to the public as a publication upon completion of the entire clinical trial.

## Discussion

PI has been used to evaluate the effect of brachial plexus, sciatic nerve, stellate ganglion in adults, and sacral canal block in children [18–20]. Sebastiani Anne et al. reported that PI increased in successful intermuscular sulcus nerve block, which can be used as an indicator of successful block in conscious patients [19]. Abdelnasser A et al. studied 77 adult patients undergoing elective orthopedic surgery via ultrasound-guided supraclavicular brachial plexus block. They found that the PI value of the blocked limb increased compared with the baseline value, and the ratio of PI values was higher than that of the limbs on the unblocked side at all time points. In addition, when the PI increment and PI ratio are 3.3 and 1.4 respectively at 10 min after injection as the limit to determine the success of the block, both the sensitivity and specificity for the success of the block were 100% [15]. When PI is used to evaluate the effect of sacral canal block under ketamine anesthesia in children, it is an earlier, more objective, and sensitive indicator than acupuncture sensation and cremasteric reflex [18]. However, the studies mentioned above have certain limitations. The effect of nerve block is not all-or-none. Abdelnasser A et al. did not analyze the cases of block failure and did not record the changes in PI values of partial block [15]. Since the brachial plexus is a cluster of nerves, some nerves are relatively difficult to block with different blocking methods. Therefore, a suitable evaluation method should be able to accurately assess the block's complete, partial, and failure.

The pediatric nerve block is mainly performed under sedation or general anesthesia [21, 22]. Sevoflurane and propofol are commonly used for induction and maintenance of general anesthesia in children [23, 24], but both have a vasodilator effect. Sebastiani Anne found that PI values of the two limbs were not significantly different but relatively higher than baseline values after 25 min of intermuscular sulcus block and sevoflurane inhaled for maintenance. Sevoflurane is considered to increase PI value by increasing blood flow of the muscle tissue on the unblocked side, and the PI value of the blocked side does not increase, which means that vasodilatation has reached the maximum after nerve block [19]. Park SG et al. found that the PI value increased after propofol anesthesia induction and sevoflurane anesthesia maintenance in patients without regional block [25]. Both propofol and sevoflurane general anesthesia affects the PI value. There is still no study about PI changes after brachial plexus nerve block after sevoflurane or propofol anesthesia induction in children. We speculate that the changes of PI after brachial plexus block may exist in different periods after the administration of sevoflurane or propofol. For example, when the vasodilator effect of propofol reaches its peak, the PI may have increased to the highest value. At this time, PI may not increase further after nerve block. However, PI values will change differently following the metabolism of anesthetics on the blocked and non-blocked sides. Moreover, the surgery for upper limb fractures could always be completed in about 1 h in children. And anesthesia and analgesia effect last 6–8 h after nerve block with ropivacaine. We will monitor the PI values of both sides after nerve block at the beginning and the end of the surgery. The influence of general anesthetics on PI values will be excluded after children wake up.

In conclusion, we intend to evaluate the prediction effect of PI on supraclavicular brachial plexus block after the induction of general anesthesia with sevoflurane or propofol in children, which could be used as a clue to evaluating the requirements of postoperative analgesics in children.

### Trial status

1. Protocol version number and date: Ethics Approval No. 2020-S134 on 15 July 2020
2. Date recruitment began: May 2021
3. Approximate date when recruitment will complete: December 2022

## Data Availability

The principal investigators of this study have access to the data. The datasets analyzed during the current study are available from the corresponding author upon reasonable request. Any data required to support the protocol can be supplied on request.

## Abbreviations

ASA: American Society of Anesthesiologists
BMI: Body mass index
BPB: Brachial plexus block
MAC: Minimal alveolar concentration
PACU: Post anesthesia care unit
PI: Perfusion index
PRO: Propofol
RCT: Randomized controlled trial
SEV: Sevoflurane
SCB: Supraclavicular brachial plexus block
